# Impact of COVID-19 on Guillain Barre Syndrome (GBS) insights from an Iranian referral center

**DOI:** 10.3389/fneur.2025.1565912

**Published:** 2025-05-07

**Authors:** Mohammadali Arami, Rezafarhad Manteghifasaei, Mohammad Karimi, Puya Zandi, Mobina Javadi

**Affiliations:** ^1^Department of Neurology, Milad General Hospital, Tehran, Iran; ^2^Faculty of Medicine, Shahid Beheshti University of Medical Sciences, Tehran, Iran

**Keywords:** Guillain-Barré syndrome, COVID-19, COVID 19 vaccine complications, acute motor axonal neuropathy (AMAN), acute inflammatory demyelinating polyneuropathy (AIDP)

## Abstract

**Background:**

The COVID-19 pandemic has raised concerns about various neurological complications, including Guillain-Barré syndrome (GBS). Understanding the characteristics and incidence of GBS during this period is crucial for clinical management and public health response.

**Objective:**

This study aims to analyze the characteristics of GBS cases reported at an Iranian referral center during the COVID-19 pandemic and assess the relationship between GBS, COVID-19 infection, and vaccination.

**Methods:**

We conducted a retrospective analysis of GBS cases referred to our center between 2018 and 2023. Clinical data, including demographic information, neurophysiological subtypes, COVID-19 and vaccination associated cases and outcomes were collected and analyzed. The frequency of GBS admission during pandemic compared with previous years. Characteristics of COVID-19 associated GBS and those vaccinated for COVID-19 was compared.

**Results:**

A total of 334 patients were included to study. Our analysis revealed a notable increase in GBS cases during the pandemic. The incidence of vaccine-related GBS was significantly lower than that of GBS related to COVID-19 infection. Additionally, GBS patients diagnosed with COVID-19 presented more severe symptoms compared to those who developed GBS after vaccination.

**Conclusion:**

Although the incidence of GBS increased during the COVID-19 pandemic at our center, it remains a rare complication of both COVID-19 and its vaccination. These findings highlight the need for ongoing surveillance of neurological complications during infectious disease outbreaks and vaccination campaigns.

## Introduction

The Iranian Ministry of Health officially announced the first case of COVID-19 in Iran in February 2019. Prior to this announcement, there had been reports of suspicious cases; however, due to the lack of definitive diagnostic facilities and insufficient familiarity among doctors, these cases were misdiagnosed and treated as influenza. The global spread of the virus and the reporting of positive cases in neighboring countries heightened awareness of the disease. Additionally, the identification of specific characteristics in the CT scans of COVID-19 patients facilitated the diagnosis of severe cases, leading to the official declaration of the pandemic’s arrival in Iran (1, 2).

In 2020 and 2021, the COVID-19 virus infected a significant portion of the population, resulting in several peaks of transmission. Although there was another peak in 2022, it was associated with fewer fatalities. By May 2023, the incidence of the disease had significantly decreased in Iran, marking the end of the pandemic phase.

Vaccination efforts began in late 2020 and continued through 2021 and 2022. The most widely available vaccines against SARS-CoV-2 in Iran included AstraZeneca, Sinopharm, and Sputnik V, with Sinopharm being the most commonly administered (3).

GBS is an acute inflammatory polyneuropathy characterized by humoral and cellular immune responses that lead to the destruction of the myelin sheath surrounding the axons of peripheral nerves. GBS encompasses several variants that differ in their pathophysiology, clinical presentations, and outcomes; these are primarily classified into demyelinating and axonal subtypes. For instance, immune reactions targeting epitopes on Schwann cell membranes or myelin result in demyelinating neuropathy, whereas reactions against axonal membrane antigens lead to the acute axonal form of the syndrome (4). Koike et al. demonstrated that initial macrophage-associated demyelinating lesions were primarily located at internodes and nodal regions. The sites of macrophage-initiated phagocytosis of myelin may correlate with areas of complement deposition in certain patients with acute inflammatory demyelinating polyneuropathy (AIDP) (5). The axonal form of GBS is increasingly recognized as a significant subtype, distinguished by the localization of at least one target antigen on the axolemma or peri-axonal space, rather than the myelin sheath itself. The remarkable sparing of sensory fibers in cases with the acute motor axonal neuropathy (AMAN) pattern could indicate an immune attack on epitopes predominantly found on motor fibers. In contrast, cases characterized by degeneration of both motor and sensory fibers, known as acute motor and sensory axonal neuropathy (AMSAN), may involve a more generalized axonal antigen (6).

Case reports of the Miller Fisher syndrome (MFS) associated with COVID-19 have been published. In most instances, the disease is mild and transient. Additionally, cases of the MFS have been reported following COVID-19 vaccination (7). Symptoms of MFS may emerge simultaneously with COVID-19 symptoms or even weeks after the infection, suggesting that molecular mimicry and/or bystander activation may begin during the disease’s incubation phase.

While the majority of patients with MFS are positive for GQ1b antibodies, a systematic review found that this is often not the case in those associated with COVID-19. interestingly, anti-GQ1b antibody positivity is detected more frequently in COVID-19 vaccination associated MFS (7). A recent systematic review indicated that ganglioside autoantibodies, which are important biomarkers in MFS, are not effective in distinguishing the COVID-19-associated variant (8). This raises the possibility that arginylglycylaspartic acid (RGD) may serve as an alternative receptor for cell adhesion in the neuropathogenesis of COVID-19-associated MFS. Therefore, it appears that distinct targets and different immune-mediated mechanisms may underlie this condition (9).

Several infectious agents, such as *Campylobacter jejuni* and *Haemophilus influenzae*, have been linked to the onset of this syndrome (10–12). From the onset of the pandemic, COVID-19 has also been associated with effects on the peripheral nervous system in some patients, presenting clinical features similar to GBS. However, questions remain regarding the prevalence of GBS among COVID-19 patients, as well as the clinical differences and mortality rates associated with GBS. With sufficient time having passed since the pandemic’s peak, we can now analyze the data more accurately to address these questions.

## Materials and methods

Milad General Hospital is one of the largest hospitals in Tehran, the capital of Iran, which has a population of over 16 million. This hospital serves a substantial population from Tehran and neighboring cities and is recognized as a well-equipped referral center. More than ten neurologists work at Milad General Hospital, which features advanced emergency departments, an intensive care unit (ICU), and plasmapheresis facilities.

In this study, we reviewed the medical records of patients diagnosed with GBS who were admitted to our center during the COVID-19 pandemic, collecting essential data for analysis. To accurately compare the prevalence of GBS, we also examined data from patients admitted in 2018 and during the first 9 months of 2019 and 2023.

We analyzed several key features, including the demographic characteristics of the patients, the interval between the onset of symptoms and COVID-19 infection, the severity of the disease, the type of nerves pathology based on neurophysiological testing, vaccination history, and the interval between vaccination and the onset of GBS symptoms. The inclusion criteria for the study required evidence of COVID-19 infection, which included fever, hypoxemia as indicated by pulse oximetry, and respiratory symptoms, along with COVID-19-compatible lung involvement on chest CT scans. Additionally, close contact with a confirmed COVID-19 patient and/or a positive COVID-19 PCR test were also necessary criteria, all validated by infectious disease specialists. The diagnosis of GBS was made based on established criteria developed by the National Institute of Neurological Disorders and Stroke (NINDS) (13).

According to neurophysiological studies, patients were classified into three types: Acute Inflammatory Demyelinating Polyneuropathy (AIDP), Acute Motor Sensory Axonal Polyneuropathy (AMSAN), and Acute Motor Axonal Polyneuropathy (AMAN). For electrophysiological classification, we applied Uncini’s criteria (14). In some patients, we conducted a second electrophysiological study to enhance classification accuracy. We routinely analyze the cerebrospinal fluid (CSF) of all patients and perform additional investigations if results are incongruent with a GBS diagnosis. CSF analysis for all studied patients was consistent with the diagnosis of GBS.

Patients were categorized into three groups: Group 1 consisted of GBS patients without recent COVID-19 infection; Group 2 included patients with GBS occurring a few days or weeks after COVID-19 infection (COVID-19 associated GBS); and Group 3 comprised patients with GBS developing a few days or weeks after vaccination (post-COVID-19 GBS). We further classified patients based on disease severity into two groups: those without severe laryngeal/respiratory involvement who did not require intubation or mechanical ventilation, and those in the severe group who were intubated and placed on a ventilator. The treatment protocols for the patients in all three groups remained consistent, encompassing respiratory support, rehabilitation care, plasma exchange, and intravenous immunoglobulin (IVIG) therapy, with some patients receiving both treatments.

Data were analyzed using SPSS version 26 statistical software.

## Results

A total of 334 patients were studied, comprising 149 females (44.6%) and 185 males (55.4%). The mean age of the patients was 51.38 years, with a range from 6 to 95 years. In terms of classification based on Uncini’s criteria, 217 patients (65%) had AIDP type, 84 patients (25.1%) had AMSAN type, and 33 patients (9.9%) had AMAN type. Regarding disease severity, 279 patients (83.5%) presented with mild to moderate forms, while 55 patients (16.5%) had severe forms (Table 1).

**Table 1:** Clinical characteristics.

Patients’ characteristics	(*n* = 334)
Demographic characteristics	
Age	51.38y (6–95)
Sex (M/F)	185/149 (1.24:1)
Disease type (%)	
AIDP	217 (65%)
AMAN	33 (9.9%)
AMSAN	84 (25.1%)
GBS clinical severity	
Mild to moderate	279 (83.5%)
Severe	55 (16.5%)
Time interval (days)	
For post-COVID-19 vaccination GBS	16.7 ± 6.4
For COVID-19 associated GBS	16.4 ± 8.3

AIDP, acute inflammatory demyelinating polyneuropathy; AMAN, acute motor axonal neuropathy; AMSAN, acute motor-sensory axonal neuropathy.

**Figure 1:**
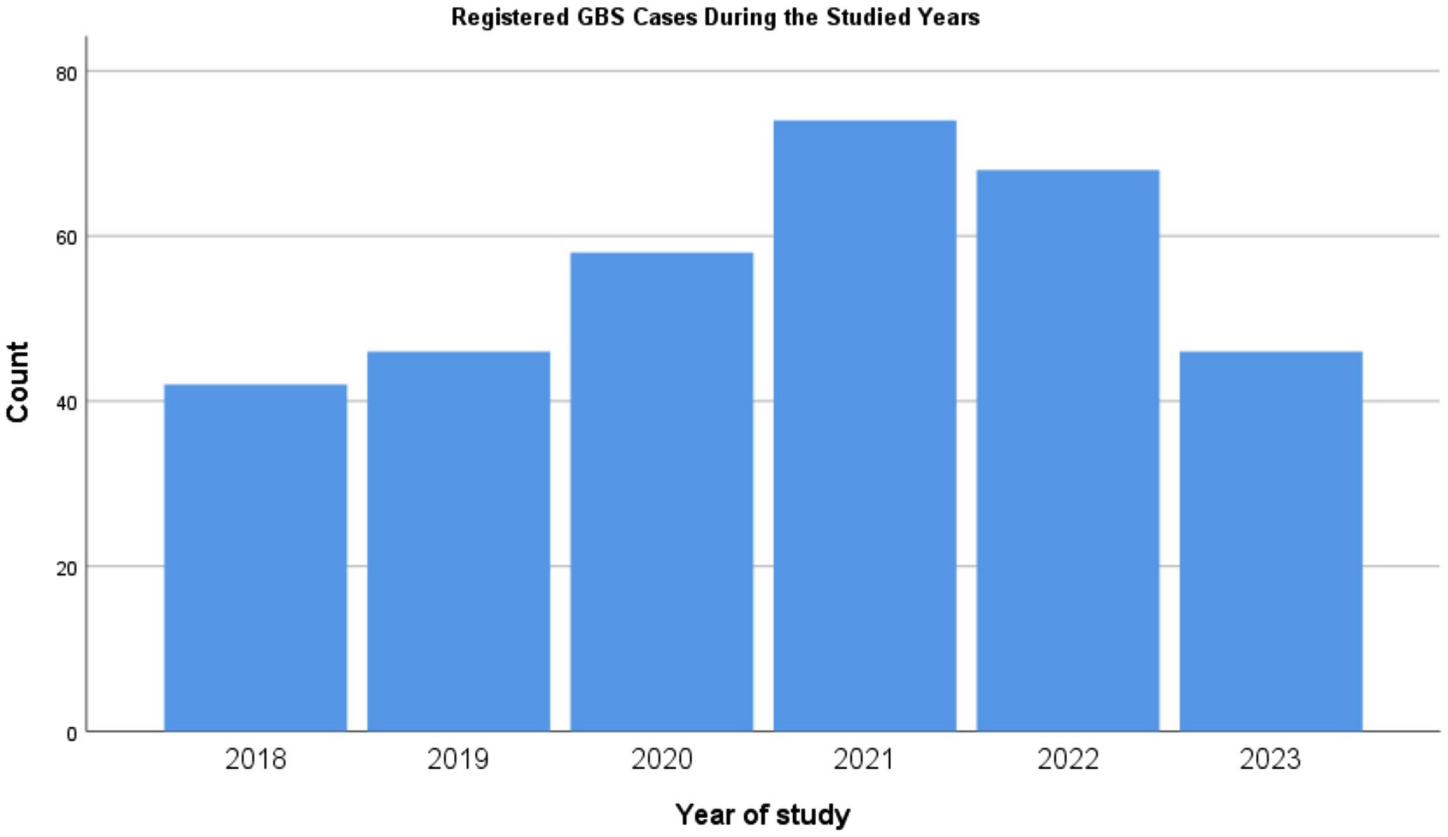
Diagram illustrating Guillain-Barré syndrome (GBS) cases registered during the pre-COVID-19 period and throughout the pandemic years. The data indicate that as the number of COVID-19 cases peaked in Iran, there was a corresponding increase in the number of hospitalized GBS cases.

Our analysis indicates a significant increase in the incidence of GBS during the pandemic years (*p* < 0.008), with a return to pre-pandemic levels following the end of the pandemic. Figure 1 illustrates the changes in incidence over the study period.

**Figure 2:**
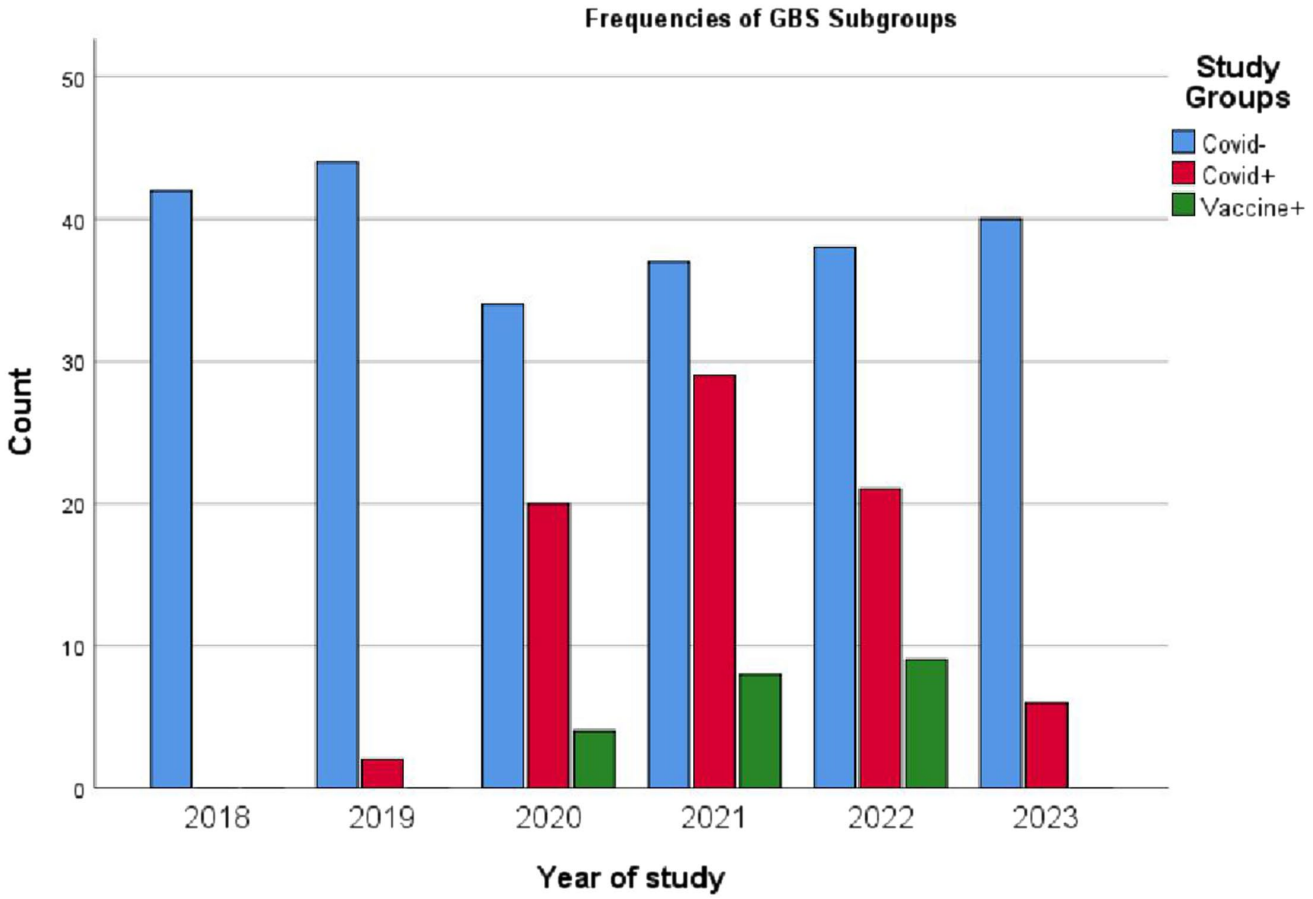
Frequency of three subgroups of Guillain-Barré syndrome (GBS) cases across each year of the study. Subgroups include: Covid−, GBS cases without concurrent COVID-19 infection; Covid+, GBS patients with COVID-19 infection; Vaccine+, GBS cases occurring post-vaccination. The data indicate a significantly lower number of post-COVID-19 vaccination GBS cases compared to GBS cases associated with COVID-19 infection, despite millions of Iranians having received the COVID-19 vaccine.

Table 2 presents the distribution of the three groups of GBS cases across the years studied. When considering 2018 as a control year, the table shows the proportion of GBS cases related to COVID-19 infection and vaccination compared to those without any connection to the pandemic. Overall, 235 cases (70.4%) of the 334 GBS patients had no evidence of COVID-19. Among them, 78 patients (23.4%) had a confirmed COVID-19 infection, and 21 patients (6.3%) developed GBS following vaccination. These figures are detailed separately for each year in Table 2. Figure 2 also illustrating these findings.

**Table 2:** GBS subgroups frequencies.

Year	All	Covid-	Covid+	Vaccine+
2018	42	42 (100%)	0	0
2019	46	44 (95.7%)	2 (4–3%)	0
2020	58	34 (58.6%)	20 (34.5%)	4 (6.9%)
2021	74	37 (50%)	29 (39.2%)	8 (10.8%)
2022	68	38 (55.9%)	21 (30.9%)	9 (13.2%)
2023	46	40 (87%)	6 (13%)	0
All	344	235 (70.4%)	78 (23.4%)	21 (6.3%)

The mean ages of patients in the three groups were 50.8 ± 18.23 years, 52.7 ± 18.7 years, and 53.2 ± 13.5 years, respectively. Statistical analysis revealed no significant age differences between the three groups (*p* < 0.6).

CSF protein levels were above the normal range (15–45 mg/dL) in 305 (91%) patients, with a median cell count of 3.1 cells/μL (range: 0–6 cells/μL). No significant differences were observed in protein levels (*p* < 0.7) or CSF cell counts (*p* < 0.6) between patients with COVID-19 associated GBS, those following vaccination and those without any relation to COVID-19. We did not include patients with the MFS among hospitalized patients, so anti-GQ-1b was not measured.

The mean time interval between contracting COVID-19 and the onset of GBS was 16.4 ± 8.3 days, this time was 16.7 ± 6.4 days for post-COVID-19 vaccination and the onset of GBS. Also, Of the 21 post-COVID-19 vaccination GBS patients, 11 patients had the Sinopharm COVID-19 vaccine (inactivated virus). 4 patients had the Oxford-AstraZeneca COVID-19 vaccine (viral vector vaccine) and 6 patients had the COViran-Barekat COVID-19 vaccine (inactivated virus). GBS occurred after the first dose of vaccination in 11 patients (52%) and 10 patients (48%) suffered GBS after second dose.

**Figure 3:**
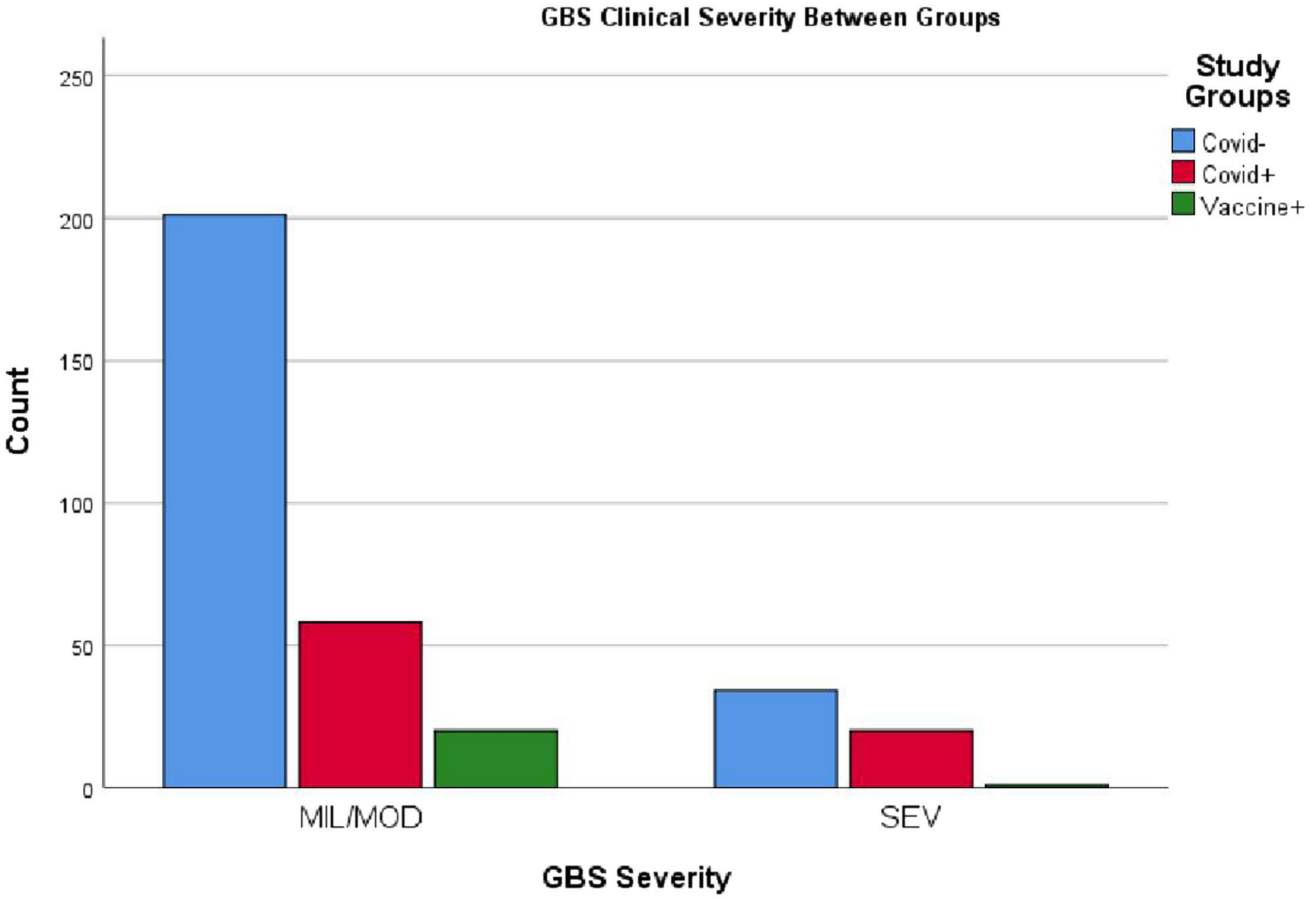
Clinical status of patients across the three GBS subgroups. MIL/MOD, mild and moderate conditions; SEV, severe critical condition. The figure illustrates that severe cases were more prevalent in the GBS with COVID-19 group, while the majority of patients in the post-vaccination GBS group presented with mild to moderate forms. Please refer to the text for further details.

Data analysis indicated that COVID-19 associated GBS patients experienced higher disease severity and mortality compared to the other two groups. In this cohort, 20 out of 78 patients (25.6%) developed severe disease, which is statistically significant (*p* < 0.02). It is important to note that patients from 2018 and the early months of 2019 are included in this calculation, a period before COVID-19 had spread to countries such as Iran. Among the 21 patients with post-vaccination GBS, only one (4.8%) developed severe disease. Of the 334 GBS patients, 11 died, with the highest number of fatalities occurring among those with COVID-19. The mortality rates were 2.6% in the GBS group without COVID-19 or vaccination and 8.9% in the COVID-19 associated GBS, and there were no deaths in the post-vaccination GBS group (*p* < 0.005). No significant association was observed regarding GBS severity among the three study groups (*p* > 0.6) (see Figure 3).

**Figure 4:**
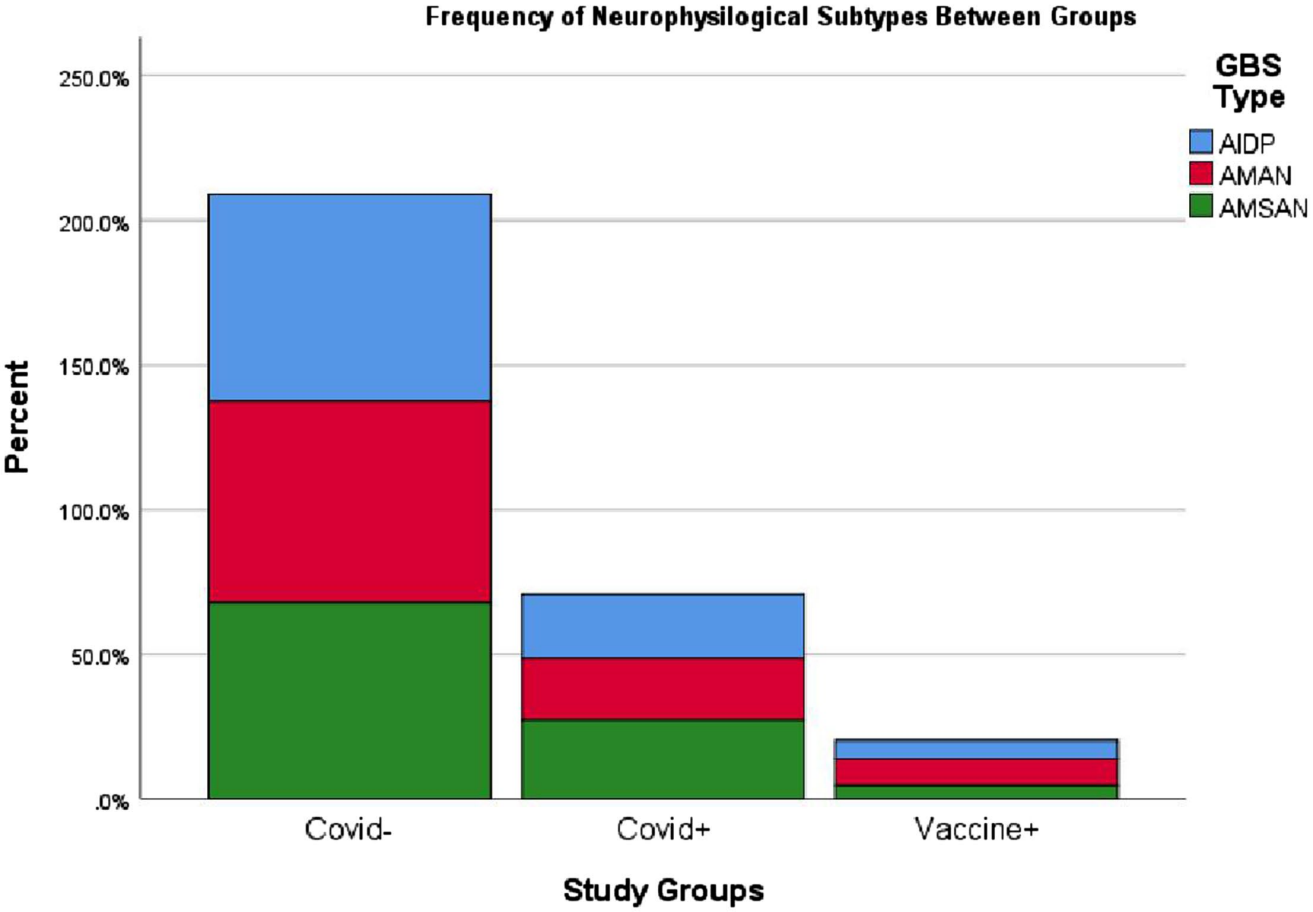
Neurophysiological subtypes between each GBS subgroups. AIDP, acute inflammatory demyelinating polyneuropathy; AMAN, acute motor axonal neuropathy; AMSAN, acute motor-sensory axonal neuropathy. As you can see, the distribution of neurophysiological types of GBS is almost similar among the 3 patient groups, which can be interpreted as the presence of the same pathophysiology in all three conditions.

Table 3 compares the frequency of neurophysiological types of GBS among the study groups. No significant differences were observed in the distribution of neurophysiological types among patients with COVID-19-associated GBS, post-COVID-19 vaccination GBS and those who had never contracted COVID-19 (*p* > 0.6). Figure 4 provides a visual representation of these findings.

**Table 3:** GBS neurophysiological types frequencies.

Year	All	Covid-	Covid+	Vaccine+
AIDP	217 (65%)	155 (66%)	48 (61.5%)	14 (66.7%)
AMAN	33 (9.9%)	23 (9.8%)	7 (9%)	3 (14.3%)
AMSAN	84 (25.1%)	57 (24.3%)	23 (29.5%)	4 (19%)

AIDP, acute inflammatory demyelinating polyneuropathy; AMAN, acute motor axonal neuropathy; AMSAN, acute motor-sensory axonal neuropathy.

## Discussion

Analysis of data from our center confirms a significant increase in GBS during the COVID-19 pandemic. Known infectious agents, such as *Campylobacter jejuni*, arboviruses, and urinary tract infections, are established causes of GBS; the COVID-19 virus has also been added to the list of underlying factors associated with this syndrome. While the prevalence of GBS among patients with *Campylobacter jejuni* or *Haemophilus influenzae* remains unclear, the widespread transmission of COVID-19, which infected millions in just a few months, suggests that GBS is a rare complication of COVID-19, albeit more common than other peripheral nervous system complications linked to the virus. In COVID-19 admissions, the reported frequency of GBS was 0.07%, according to Sharma et al. (15), while Censi et al. estimated an incidence of 0.13 per 1,000 among the SARS-CoV-2-infected population in Italy (16).

SARS-CoV-2 can trigger GBS via several pathways like direct neurotropism and neurovirulence, microvascular dysfunction and oxidative stress, immune system disruption, molecular mimicry, and autoantibody production (17).

As COVID-19 vaccination began in Iran in late 2020, the rise in documented GBS cases in that year was primarily associated with COVID-19 infection. The notable increase in registered GBS cases in 2021 compared to 2022 may also reflect the reporting of cases related to vaccination. Our data analysis indicates that the incidence of post-COVID Vaccination GBS is lower than that associated with COVID-19 infection. Despite the rapid and widespread vaccination efforts in Iran since late 2020, the recorded cases of post-COVID Vaccination GBS demonstrate that it remains a rare complication. Moreover, the high frequency of mild to moderate GBS and the low mortality rates in this group support the notion that complications following vaccination are not only rare, but also mild, with most patients recovering fully. In a systematic review conducted by Abolmaali et al., AstraZeneca was the most-reported vaccine associated with GBS followed by Pfizer. GBS occurred after the first dose of vaccination in 79.5% of cases and the mean time interval between vaccination and GBS occurrence was 13.9 ± 7.4 days (18). Since the rates of different vaccines administered separately in the Iranian population are not available and given the small number of Guillain-Barré cases post-vaccination, we cannot determine which vaccine type is more commonly associated with the occurrence of GBS.

Miao et al. aimed to retrospectively analyze reported GBS cases that occurred after COVID-19 vaccination. They detailed analysis of 60 case reports revealed that post-COVID-19 vaccination GBS occurred mostly after the first dose of the vaccination (54 cases, 90%) and was common for DNA vaccination (38 cases, 63%), common in middle-aged and elderly people (mean age: 54.5 years), and also common in men (36 cases, 60%). The mean time from vaccination to onset was 12.3 days. The classical GBS (31 cases, 52%) was the major clinical classification and the AIDP subtype (37 cases, 71%) was the major neurophysiological subtype (19). Censi et al. reported that the GBS risk was 2.6 times increased with the first dose and Adenovirus-vectored vaccines showed a 2.4 times increased risk of GBS that was about seven times higher compared with mRNA-based vaccines (20). The differences observed between our results and these studies could be related to the type of vaccines used, because in Iran, most injectable vaccines were inactivated virus types.

In contrast, COVID-19 associated GBS cases tend to present with more severe clinical conditions and higher mortality rates. A review of our patient records, particularly among those who succumbed to the illness, revealed that these patients typically exhibited extensive pulmonary involvement due to COVID-19. Most did not directly visit the emergency room or neurology clinic; instead, they were admitted to pulmonary or infectious disease departments and later consulted with a neurologist. According to national COVID-19 protocols, patients with unstable clinical status or respiratory distress were prioritized for admission. Conversely, patients developing post-COVID vaccination GBS generally accessed the neurology clinic as outpatients and were often treated in that setting.

Furthermore, the similar and statistically non-significant distribution of neurophysiological variants of GBS among the three patient groups indirectly suggests that peripheral nerve damage pathology is similar across all groups. The most common variant of GBS observed in Bentley et al. study was acute inflammatory demyelinating polyneuropathy (AIDP) and patient mortality among the cases was 10.9% (21). Some researchers consider axonal damage to be a factor for poor prognosis (22, 23). Additionally, Sharma et al. reported that GBS cases associated with COVID-19 exhibited significantly higher mortality rates (12.2% compared to 3%, *p* < 0.001) compared to cases without COVID-19 (15).

Although sporadic cases of MFS have been reported from various countries, we did not observe any among our patients. This may be attributed to a lower prevalence of this variant in Iran, although there is no published reference to support this claim except for a review article by Poyraz et al., which reported only one case from Iran (24). Another possibility is that the clinical conditions of patients with this variant are mild, which could explain the lack of referrals and hospitalizations to our center.

Our study has some limitations to consider. Early in the pandemic, diagnoses could be less accurate, as limited access to PCR testing meant that approximately 18% of our patients had not been tested. For these patients, we relied on clinical criteria and lung CT scans to support their diagnoses. Additionally, the pandemic’s impact on hospitals may have led to underdiagnosis of mild COVID-19 cases and Guillain-Barré syndrome (GBS). Follow-up care for GBS patients related to COVID-19 was also less consistent due to quarantine restrictions. Consequently, we could not fully capture the progression of COVID-19 associated GBS and post-vaccination GBS, focusing primarily on mortality observed during the acute phase.

## Conclusion

Despite a significant increase in registered cases of GBS at our center during the COVID-19 pandemic, this syndrome remains a rare complication of both COVID-19 and COVID-19 vaccination. This assessment takes into account the high incidence of the disease and the rapid vaccination of the population over a short period.

## Data Availability

The original contributions presented in the study are included in the article/supplementary material, further inquiries can be directed to the corresponding author.
